# Measuring the continuing care needs of inpatients in rural China

**DOI:** 10.1186/s12913-024-10729-2

**Published:** 2024-03-07

**Authors:** Haoran Liu, Zhifan Wang, Juan Hu, Qiushuang Xu, Lei Yang, Weiyan Jian

**Affiliations:** 1 Department of Health Policy and Management, School of Public Health, Peking University, Beijing, China; 2 Henan Honliv Hospital, Changyuan, China

**Keywords:** Readiness for hospital discharge, Readiness for hospital discharge scale (RHDS), Continuing care needs, Hospitalized patient, Rural China

## Abstract

**Background:**

International experience shows that the suitability of a high-performance healthcare system for its given purposes is reflected in its ability to provide a continuum of services that match the changing health status of the given population. Although many low- and middle-income countries have sought to bring movement away from hospital-centered and towards patient-centered healthcare, such efforts have often had poor results, and one of the major reasons for this is the inability to accurately identify which inpatients need continuing care and what kind of continuing of care is needed.

**Objectives:**

To measure and assess the continuing care needs of discharged patients and its influencing factors in rural China.

**Methods:**

Data were obtained from the hospital database of Medical Center M in County Z from May to July 2022. County Z is a county of 1 million people in central China. The database includes basic patient information, disease-related information, and information on readiness for hospital discharge. Factors related to the need for continuing care were included in the analysis. The Readiness for Hospital Discharge Scale was used to assess the need for continuing care. The statistical data are expressed in terms of both frequency and composition ratio. Finally, linear regression was used to analyze the factors influencing the need for continuing care.

**Results:**

The analysis included a total of 3,791 patients, 123 of whom (3.25%) had continuing nursing needs. The need of continuing nursing was related to patients’ age group, mode of admission, occupation and major diagnostic categories (*P* < 0.05).

**Conclusions:**

Developing continuing care is an important initiative for bridging the fragmentation of health services, and an appropriate supply system for continuing care, interconnected with inpatient services, should be established in rural areas in China as soon as possible. And provide more appropriate care for patients in need.

**Supplementary Information:**

The online version contains supplementary material available at 10.1186/s12913-024-10729-2.

## Background

International experience shows that the ability to provide continuing care that matches the changing health status of the given population is an important sign of that healthcare system’s effectiveness [1, 2]. Related nursing concepts include continuing care, transitional care, long term care. Continuing care has a similar connotation to transitional care, but transitional care focuses more on helping patients transition from an in-hospital to an out-of-hospital environment, while continuing care focuses on the continuation of care after discharge. Long Term Care is a policy in China to provide long-term medical care and living care to patients who live at home or in nursing homes and are unable to take care of themselves.

After the patient is discharged, the medical institution provides the continuing nursing care to the patient. Internationally, countries have taken various measures in continuing care. For example, the National University Health System in Singapore developed a transitional care program [3] with the aim of assisting elderly patients with multiple chronic diseases and walking limitations, and family caregivers, to improve quality of care and reduce hospitalization rates. Similarly, the acute care for elderly program [4] is another model of care geared towards elderly orthopedic patients in the United States. The German long-term care insurance [5] has also underpinned remarkable achievements after more than 20 years, and applicants of any age can receive assistance in various aspects of their daily living after examination, including hygiene, diet, mobility, and household chores. In the Japanese context, long-term care insurance [6] comprises, on the one hand, assistance in daily life in relation to clothing, food, housing, and transportation, and, on the other, medical treatment, nursing care, and rehabilitation training. China has also established long-term care insurance [7].

Despite the evidence pointing to the benefits of such a distributed model of healthcare provision, healthcare systems in many low- and middle-income countries (LMICs), including China, remain hospital-centered, with a disconnect between primary care and secondary and tertiary care [8, 9]. Indeed, there is evidence that even out-of-hospital rehabilitation is disconnected from inpatient services for post-discharge inpatients [10]. A study by Cody Cichowitz et al. conducted in a South African hospital found that more than half of HIV patients did not access healthcare within 2 months after discharge [11]. This resulted in missed opportunities to prevent hospitalization through changing treatment, to increase support and to secure an early diagnosis, leading to high rates of avoidable readmissions. In their study conducted in a hospital in Hebei in China, Xu Yan et al. [12] report that patients discharged with peripherally inserted central catheter experienced significantly higher rates of out-of-hospital infections than in-hospital ones and did not receive adequate educational guidance. Yang Siyao [13], conducted in a hospital in Liaoning, found that some patients discharged from the hospital on home peritoneal dialysis were not given appropriate healthcare guidance and were consequently more likely to experience problems resulting in negative dialysis-related outcomes. Similar results have been obtained in several other such studies [14, 15]. One of the major reasons for this is the inability to accurately capture which inpatients need continuing care and what kind of continuing care is needed [16, 17], with a lack of clarity about patients’ continuing care needs preventing the allocation of human-based and other forms of inputs as a consequence [18, 19].

Thus, assessing the patient’s status at discharge and the need for ongoing care can help reduce these adverse events. At the same time, the patients’ preparation for discharge was evaluated on the day of discharge, and the patients did not receive the same nursing care after discharge, so most patients still had the same problems and status as on the day of discharge. Therefore, it can be used as an indicator to assess patients’ continuing care needs. Therefore, assessing the patient’s status at discharge and the need for continuing care can help to reduce these adverse events, which can be used as an indicator to assess patients’ continuing care needs. Readiness for hospital discharge is the ability of a patient to recover further after leaving the hospital and to return to his or her family and society, potentially with the aid of a caregiver, following a comprehensive physical, psychological and social assessment by healthcare workers [20]. Discharge readiness is used to measure whether a patient needs continuing care. Readiness for hospital discharge is an important predictor of quality of recovery, and accurate assessment of readiness for discharge can prevent premature discharge, reduce the incidence of post-discharge complications and readmission rates, and save on healthcare resources and costs [20, 21].

This study was conducted on data associated with a central Chinese county of 1 million people (County Z, which we refer to “County Z” because the data provider does not want to disclose the name of the county) in order to measure the probability of patients requiring continuing care and factors influencing the need for such continuing care. Specific care needs for patients discharged from local medical centers were then analyzed. This analysis is useful not only for those involved in developing systems of continuing care in rural China, but also for those seeking evidence to support the optimization of health care systems in other LMICs.

## Research subject and methodology

### Study design

Medical Center M has 1,500 open beds and provides most of the healthcare services offered in County Z. All patients presenting with severe cases in County Z are admitted to this medical center, meaning that there is a high concentration of local inpatients with a need for post-discharge continuing care. Medical Center M launched a continuing care program in 2019 to provide care to residents in or near the host county. The program covers a number of services, including basic care, specialty care, and health guidance, all of which are intended to meet the needs of discharged patients in terms of continuing care services. The vast majority of such services must be provided by professional medical and nursing staff, with patients and families not normally being able to do this. The program is provided through charging fees for continuing care services, with prices set to be the same or slightly lower than the price of services in the same tier of medical institutions.

The Regional Medical Center uses Readiness for Hospital Discharge Scale (RHDS) to assess whether patients need further care after discharge (Table 1). The RHDS was developed by Weiss et al. [20] in 2006, following which it was translated into Chinese by Youhua Lin [22] in Taiwan. It not includes factors related to availability of resources in the community. Discharge readiness was originally used to assess a patient’s discharge readiness. Patients can be discharged if they are well prepared, otherwise discharge is not recommended. Medical Center M modified the form after in-hospital expert evaluation to assess patients’ needs for nursing services after discharge. The nurse assessed patients at discharge on the basis of Table 1. This assessment is routine and the assessment process is standardized. And combined with patients still exist in the nursing problems, to assess the needs of patients for continued care. Assessment scores are available and all data are secondhand from hospital databases.

**Table 1:** Readiness for hospital discharge scale

Evaluating standard	Scores	Items
Awareness	0	Sober
	1	Hypersomnia
	2	Lethargy
	3	Coma
Mobility	0	Completely autonomous activity
	1	Less than 50% of waking time is confined to the bed or chair
	2	More than 51% of waking time is confined to the bed or chair
	3	Unable to provide self-care, completely confined to the bed or chair
Urination	0	Normal
	1	Occasional incontinence (≤ 2 times)
	2	Incontinence (≥ 3 times/day)
	3	Indwelling urethral tube or cystostomy tube
Stool	0	Normal
	1	Occasional incontinence or auxiliary defecation (≤ 2 times)
	2	Incontinence (≥ 3 times/day)
	3	Artificial anus
Patient’s self-care/family’s caregiving ability	0	Having the ability to self-care
	1	Patients cannot self-care, family members can help complete
	2	Patients and their families lack some capacity to care but can seek resources
	3	Patients and their families lack the capacity to care for patients and seek resources
Diet	0	Normal
	1	Dysphagia, can be taken orally
	2	Dysphagia, can be feeding orally (IOE)
	3	Indwelling gastric tube, jejunal tube, gastrostomy

In the case of the patients whose data made up the database considered for this study, each patient was assessed with a scale within four hours of scheduled discharge. The total possible obtainable score is 18; the higher the patient’s score, the lesser the readiness for discharge and the greater the need for continuing care. The experts in the hospital agreed that scores ≥ 8 were considered as indicating that the patient was poorly prepared for discharge and required further nursing-based services following discharge.

### Data sources

For this study, a database was obtained containing care information relating to patients discharged between May and July 2022 from Medical Center M in County Z. After outliers and missing values were deleted, all desensitized data were included in the analysis. The data analyzed in this study includes basic patient information, disease-related information, and information on readiness for hospital discharge. The basic patient information included patient code, age, gender, marital status, occupation, education level, medical payment method, etc. Disease-related information included patient’s admission source, admission mode, visit purpose, primary disease, etc. Information on readiness for hospital discharge included patients’ discharge readiness score, care-related problems, whether the individual needed to receive continuing care, and specific care items.

These medical records come from the hospital’s nursing system. Basic patient information, disease-related information come from the front page of the patient’s nursing record. Nurses record this information by conducting a patient assessment on the day of the patient’s hospitalization, and it can be continuously supplemented during the patient’s hospitalization. The nurse evaluates information about readiness for hospital discharge at discharge. Doctors and nurses do not take the RHDS as a standard to measure whether patients can be discharged from hospital. Nurses use the results to guide the nursing measures that patients should receive after discharge, such as home-based gastric tube replacement and so on.

Inclusion criteria: (1) Discharged between May and July 2022. (2) Age ≥ 18 years.

Exclusion criteria: (1) Pregnant and maternal patients. (2) Not the last discharge data.

### Statistical methods

Statistical analysis was performed using Stata 16.0. Count data were expressed in terms of frequency and proportion; and linear regression analysis was used to analyze the factors influencing the need for continuing care. The model was specified as follows:

$$ y={\beta }_{0}+{\beta }_{1}{x}_{1}+\dots +{\beta }_{i}{x}_{i}+\gamma $$

$$ y$$ is the score indicating readiness for hospital discharge, $$ {\beta }_{0}$$ is the constant term, $$ {x}_{i}$$ represent, respectively, gender, age group, education level, marital status, source of admission, mode of admission, purpose of visit, occupation, medical payment method, and major diagnostic categories. $$ {\beta }_{i}$$ are the corresponding regression coefficients. $$ \gamma $$ represents the random error term. *P* < 0.05 is considered a statistically significant difference. Before conducting linear regression, the included independent variables were diagnosed for covariance to avoid multicollinearity. Tolerance was specified as > 0.1 and the variance inflation factor was specified as < 10, and there was no multicollinearity among the independent variables.

## Results

### General information

The total number of hospital discharges from May to July was 7,182. Patients with multiple hospitalizations were included in the analysis according to the assessment results at the last discharge, totaling 6,675. After processing of abnormal and missing values, 3,791 patients were ultimately included in the analysis according to the study inclusion criteria. 1,801 of these were females and 1,990 were males. The classification criteria relating to age group were based on the classification method used by the World Health Organization [23, 24], with more than half of the sample comprising middle age and young elderly people. The educational level of the discharged patients was low, with more than 80% having received only a junior high school education or below. The vast majority of patients were married. Regarding the source of admission, 76.55% of patients were admitted through non-emergency sources. Walk-in patients predominated, and more than 90% of patients did not undergo surgery during their hospitalization. 75.44% of patients were farmers, and more than half paid for their treatment using new rural cooperative medical insurance. The major diagnostic categories were classified according to ICD-10 [25], with more than 1/3 of the patients having circulatory and diseases of the digestive system; fewer patients fell into other categories for major diseases. The details are provided in Table 2.

**Table 2:** General information relating to patients discharged from the hospital between May and July 2022

Items	Categories	Cases	Proportion(%)
Gender	Female	1801	47.51
	Male	1990	52.49
Age group	Youth(≤44)	755	19.92
	Middle age(45–59)	1080	28.49
	Young elderly(60–74)	1259	33.21
	Elderly(75–89)	655	17.28
	Long-lived elderly(≥90)	42	1.11
Education level	Elementary school and below	1979	52.20
	Junior high school	1181	31.15
	Secondary or high school	384	10.13
	College	142	3.75
	Bachelor’s degree and above	105	2.77
Marital status	Never married	178	4.70
	Married	3613	95.30
Source of admission	Non-emergency sources	2902	76.55
	Emergency sources	889	23.45
Mode of admission	Walk	2804	73.96
	Stretcher	548	14.46
	Wheelchair	439	11.58
Purpose of visit	Non-surgical	3553	93.72
	Surgical	238	6.28
Occupation	Farmer	2860	75.44
	Self-employed	294	7.76
	Retired (resigned) person	140	3.69
	Worker	140	3.69
	Other	357	9.42
Medical payment method	New rural cooperative medical insurance	2116	55.82
	Basic medical insurance for urban residents	1012	26.69
	Basic medical insurance for urban workers	338	8.92
	fully self-paid	285	7.52
	Other	40	1.06
Major diagnostic categories	Certain infectious and parasitic diseases	58	1.53
	Neoplasms	233	6.15
	Diseases of the blood and blood-forming organs and certain disorders involving the immune mechanism	42	1.11
	Endocrine, nutritional and metabolic diseases	218	5.75
	Mental and behavioral disorders	16	0.42
	Diseases of the nervous system	332	8.76
	Diseases of the eye and adnexa	49	1.29
	Diseases of the ear and mastoid process	145	3.82
	Diseases of the circulatory system	1010	26.64
	Diseases of the respiratory system	216	5.70
	Diseases of the digestive system	481	12.69
	Diseases of the skin and subcutaneous tissue	16	0.42
	Diseases of the musculoskeletal system and connective tissue	99	2.61
	Diseases of the genitourinary system	256	6.75
	Congenital malformations, deformations and chromosomal abnormalities	11	0.29
	Symptoms, signs and abnormal clinical and laboratory findings, not elsewhere classified	44	1.16
	Injury, poisoning and certain other consequences of external causes	303	7.99
	Factors influencing health status and contact with health services	262	6.91

### Readiness for hospital discharge of patients

Table 3 demonstrates the discharge readiness scores of the patients. With respect to the total score, females (1.46) and males (1.51) were found to have similar scores, but females were slightly better prepared than males; with increasing age, the score was significantly higher in long-lived elderly patients than in youth patients, at 5.14 points; the scores were similar among patients with different levels of education; married patients (1.50) had a slightly higher score than never married patients (1.13); emergency admissions were significantly higher than non-emergency admissions, at 2.36 points; scores were significantly higher for patients admitted in stretchers or on wheelchairs than for patients admitted on foot, at 3.22, 2.93, and 0.92 points, respectively; scores were similar for surgical and non-surgical patients; scores were also similar for patients of different occupations and using different healthcare payment methods. The scores of the patients with different major diagnoses differed greatly. The highest score of 2.20 was for the major diagnostic category of injury, poisoning and certain other consequences of external causes; the lowest score of 0.56 was for the major diagnostic category of endocrine, nutritional and metabolic diseases.

**Table 3:** Scores of discharge readiness for patients discharged between May and July 2022

Items	Categories	Total scores (Mean + SD)	Awareness (Mean + SD)	Mobility (Mean + SD)	Urination (Mean + SD)	Stool (Mean + SD)	Patient’s self-care/family’s caregiving ability (Mean + SD)	Diet (Mean + SD)
Total		1.49 + 2.30	0.03 + 0.28	0.38 + 0.83	0.14 + 0.61	0.04 + 0.27	0.83 + 0.87	0.07 + 0.41
Gender	Female	1.46 + 2.12	0.02 + 0.21	0.37 + 0.82	0.10 + 0.51	0.03 + 0.26	0.87 + 0.86	0.06 + 0.36
	Male	1.51 + 2.45	0.04 + 0.33	0.38 + 0.84	0.18 + 0.68	0.04 + 0.29	0.79 + 0.87	0.08 + 0.45
Age group	Youth(≤44)	0.91 + 1.58	0.01 + 0.16	0.22 + 0.64	0.09 + 0.49	0.01 + 0.10	0.56 + 0.75	0.03 + 0.27
	Middle age(45–59)	1.00 + 1.70	0.01 + 0.16	0.26 + 0.70	0.08 + 0.48	0.02 + 0.19	0.60 + 0.78	0.03 + 0.28
	Young elderly(60–74)	1.58 + 2.32	0.04 + 0.31	0.39 + 0.84	0.15 + 0.61	0.05 + 0.30	0.89 + 0.84	0.07 + 0.40
	Elderly(75–89)	2.55 + 3.01	0.07 + 0.41	0.65 + 1.01	0.28 + 0.81	0.09 + 0.40	1.29 + 0.91	0.17 + 0.62
	Long-lived elderly(≥90)	5.14 + 3.51	0.24 + 0.69	1.50 + 1.13	0.62 + 1.13	0.24 + 0.53	2.29 + 0.77	0.26 + 0.80
Education level	Elementary school and below	1.78 + 2.52	0.04 + 0.30	0.46 + 0.90	0.16 + 0.64	0.05 + 0.30	0.97 + 0.90	0.10 + 0.49
	Junior high school	1.19 + 1.99	0.02 + 0.22	0.30 + 0.75	0.13 + 0.58	0.03 + 0.25	0.68 + 0.83	0.03 + 0.29
	Secondary or high school	1.27 + 2.20	0.05 + 0.35	0.33 + 0.80	0.12 + 0.55	0.04 + 0.28	0.70 + 0.79	0.04 + 0.34
	College	0.85 + 1.29	0.01 + 0.08	0.14 + 0.50	0.13 + 0.61	--	0.54 + 0.75	0.03 + 0.27
	Bachelor’s degree and above	1.02 + 1.51	0.03 + 0.29	0.22 + 0.57	0.09 + 0.50	--	0.69 + 0.79	--
Marital status	Never married	1.13 + 1.76	0.02 + 0.22	0.31 + 0.77	0.03 + 0.28	0.01 + 0.15	0.70 + 0.80	0.06 + 0.39
	Married	1.50 + 2.32	0.03 + 0.28	0.38 + 0.83	0.15 + 0.62	0.04 + 0.28	0.83 + 0.87	0.07 + 0.41
Source of admission	Non-emergency sources	1.22 + 1.89	0.02 + 0.19	0.29 + 0.71	0.12 + 0.56	0.03 + 0.24	0.73 + 0.81	0.04 + 0.30
	Emergency sources	2.36 + 3.14	0.08 + 0.46	0.67 + 1.07	0.23 + 0.75	0.08 + 0.36	1.15 + 0.97	0.16 + 0.63
Mode of admission	Walk	0.92 + 1.39	0.003 + 0.09	0.19 + 0.57	0.09 + 0.51	0.01 + 0.17	0.61 + 0.71	0.02 + 0.20
	Stretcher	3.22 + 3.67	0.14 + 0.59	0.95 + 1.21	0.33 + 0.87	0.12 + 0.43	1.43 + 1.00	0.25 + 0.80
	Wheelchair	2.93 + 2.93	0.07 + 0.41	0.88 + 1.06	0.23 + 0.72	0.11 + 0.45	1.49 + 0.93	0.14 + 0.56
Purpose of visit	Non-surgical	1.49 + 2.29	0.03 + 0.28	0.38 + 0.83	0.14 + 0.59	0.04 + 0.28	0.83 + 0.87	0.07 + 0.41
	Surgical	1.40 + 2.34	0.04 + 0.34	0.33 + 0.80	0.24 + 0.80	0.01 + 0.09	0.72 + 0.92	0.07 + 0.44
Occupation	Farmer	1.60 + 2.41	0.03 + 0.30	0.41 + 0.85	0.16 + 0.64	0.05 + 0.30	0.88 + 0.88	0.08 + 0.44
	Self-employed	1.16 + 1.95	0.02 + 0.25	0.33 + 0.76	0.08 + 0.47	0.01 + 0.14	0.67 + 0.83	0.04 + 0.35
	Retired (resigned) person	1.59 + 2.28	0.04 + 0.28	0.40 + 0.86	0.13 + 0.56	0.04 + 0.25	0.95 + 0.93	0.04 + 0.36
	Worker	1.09 + 1.98	0.03 + 0.27	0.31 + 0.78	0.15 + 0.66	0.01 + 0.17	0.56 + 0.76	0.02 + 0.25
	Other	0.91 + 1.46	0.01 + 0.16	0.22 + 0.62	0.06 + 0.42	0.01 + 0.11	0.61 + 0.76	0.01 + 0.17
Medical payment method	New rural cooperative medical insurance	1.48 + 2.23	0.02 + 0.24	0.38 + 0.82	0.15 + 0.63	0.04 + 0.29	0.82 + 0.87	0.06 + 0.39
	Basic medical insurance for urban residents	1.48 + 2.43	0.05 + 0.34	0.36 + 0.80	0.13 + 0.60	0.04 + 0.27	0.82 + 0.85	0.09 + 0.46
	Basic medical insurance for urban workers	1.38 + 2.22	0.03 + 0.25	0.30 + 0.78	0.16 + 0.65	0.04 + 0.27	0.80 + 0.88	0.05 + 0.37
	fully self-paid	1.58 + 2.36	0.04 + 0.35	0.50 + 0.94	0.09 + 0.50	0.01 + 0.17	0.88 + 0.86	0.05 + 0.39
	Other	2.33 + 2.52	0.03 + 0.16	0.85 + 1.23	0.08 + 0.35	0.05 + 0.22	1.25 + 0.95	0.08 + 0.47
Major diagnostic categories	Certain infectious and parasitic diseases	1.21 + 2.43	0.02 + 0.13	0.34 + 0.89	0.07 + 0.41	0.05 + 0.29	0.62 + 0.83	0.10 + 0.55
	Neoplasms	1.48 + 1.87	0.03 + 0.28	0.36 + 0.76	0.12 + 0.57	0.01 + 0.15	0.90 + 0.76	0.06 + 0.37
	Diseases of the blood and blood-forming organs and certain disorders involving the immune mechanism	1.69 + 2.58	0.07 + 0.46	0.45 + 0.86	0.24 + 0.79	0.05 + 0.31	0.88 + 0.89	--
	Endocrine, nutritional and metabolic diseases	0.56 + 1.22	--	0.11 + 0.49	0.03 + 0.29	0.01 + 0.14	0.40 + 0.71	0.005 + 0.07
	Mental and behavioral disorders	0.81 + 1.38	0.06 + 0.25	0.31 + 0.79	--	--	0.44 + 0.63	--
	Diseases of the nervous system	1.09 + 2.41	0.03 + 0.29	0.21 + 0.65	0.09 + 0.47	0.05 + 0.29	0.62 + 0.79	0.09 + 0.50
	Diseases of the eye and adnexa	0.57 + 0.89	--	0.06 + 0.24	--	--	0.49 + 0.74	0.02 + 0.14
	Diseases of the ear and mastoid process	0.58 + 0.74	--	0.05 + 0.22	0.02 + 0.25	--	0.50 + 0.54	0.01 + 0.08
	Diseases of the circulatory system	1.50 + 2.48	0.04 + 0.33	0.36 + 0.82	0.11 + 0.52	0.05 + 0.28	0.84 + 0.85	0.10 + 0.46
	Diseases of the respiratory system	2.14 + 3.47	0.09 + 0.44	0.56 + 1.03	0.25 + 0.77	0.08 + 0.38	0.91 + 1.06	0.25 + 0.80
	Diseases of the digestive system	1.52 + 1.86	0.03 + 0.25	0.41 + 0.78	0.10 + 0.51	0.03 + 0.24	0.92 + 0.89	0.04 + 0.29
	Diseases of the skin and subcutaneous tissue	1.19 + 1.11	--	0.25 + 0.58	--	--	0.94 + 0.68	--
	Diseases of the musculoskeletal system and connective tissue	1.86 + 1.70	--	0.71 + 0.98	--	--	1.15 + 0.90	--
	Diseases of the genitourinary system	1.79 + 2.51	0.01 + 0.19	0.33 + 0.85	0.66 + 1.23	0.04 + 0.26	0.74 + 0.89	0.02 + 0.20
	Congenital malformations, deformations and chromosomal abnormalities	0.91 + 0.94	0.000 + 0.000	0.45 + 0.93	0.000 + 0.000	0.000 + 0.000	0.45 + 0.52	0.000 + 0.000
	Symptoms, signs and abnormal clinical and laboratory findings, not elsewhere classified	1.27 + 2.51	0.000 + 0.000	0.36 + 0.87	0.11 + 0.54	0.05 + 0.21	0.68 + 0.93	0.07 + 0.45
	Injury, poisoning and certain other consequences of external causes	2.20 + 2.25	0.03 + 0.30	0.79 + 1.09	0.13 + 0.57	0.01 + 0.11	1.19 + 0.92	0.05 + 0.38
	Factors influencing health status and contact with health services	1.68 + 2.25	0.04 + 0.29	0.39 + 0.80	0.13 + 0.60	0.11 + 0.53	0.96 + 0.85	0.05 + 0.34

In the subcategories of discharge readiness score, patients had the highest scores in relation to their abilities in terms of self-care/family care, with scores ranging from 0.4 to 2.29 points and the worst readiness. The next-highest scores related to mobility, urination and stool, with moderate readiness. The lowest scores, indicating the greatest level of readiness, related to patient awareness and diet, both with scores < 0.24.

In this study, a total of 123 patients (3.25%) had a total score of ≥ 8. The final assessment of the patient before discharge was taken to represent that patient’s health status at the time of discharge, with the care problems affecting that patient at that time constituting the nursing-related needs that the patient had post-discharge. Patients with a discharge readiness score of ≥ 8 had a total of 70 nursing problems, with the sum of the top 10 nursing problems accounting for 56.2%. These were: infection, risk of falls, impaired respiratory clearance, altered ability to care for oneself in daily life, altered blood pressure, risk of impaired skin integrity, physical activity dysfunction, impaired skin integrity, risk of accidental aspiration, and electrolyte imbalance (see Appendix 1, Additional file 1). We reviewed the medical records of patients with scores greater than 8 and consulted nurses. Most of these patients were delirious or unable to take care of themselves, or were discharged with a tube. Upon discharge, the vast majority of these patients live at home and are cared for by family members or caregivers. The nurse will provide on-site care to the patient who is wearing the tube when the patient needs it, such as changing the gastric tube.

### Analysis of factors influencing readiness for discharge

Appendix 2 (see additional file 2) details the value assignment of dependent and independent variables. All influencing factors were included in the analysis and multiple linear regression was performed to analyze the factors influencing readiness for discharge (table 4). The results showed that statistical significance is evident in respect of age group, mode of admission, occupation, and major diagnostic categories (*P* < 0.05). The relevant scores were 0.572 (*P* < 0.01), 1.156 (*P* < 0.01), and 3.138 (*P* < 0.01) higher for young elderly, elderly, and long-lived elderly patients, respectively, compared to youth patients. For mode of admission, the scores were 1.727 (*P* < 0.01) and 2.252 (*P* < 0.01) higher for patients admitted in wheelchairs or on stretchers, respectively, compared to patients admitted on foot. In terms of occupation, workers’ score was 0.323 (*P* < 0.05) lower than that of farmers. In terms of major diagnostic categories, diseases of the circulatory system, with the highest number of patients, were selected as the control group. Statistical significance was found in relation to diseases of the genitourinary system, diseases of the musculoskeletal system and connective tissue, factors influencing health status and contact with health services, diseases of the respiratory system, neoplasms, injury, poisoning and certain other consequences of external causes, diseases of the digestive system, diseases of the blood and blood-forming organs and certain disorders involving the immune mechanism, congenital malformations, deformations and chromosomal abnormalities, diseases of the skin and subcutaneous tissue, with figures of 1.113 (*P* < 0.01), 0.936 (*P* < 0.01), 0.837 (*P* < 0.01), 0.828 (*P* < 0.01), 0.752 (*P* < 0.01), 0.615 (*P* < 0.01), 0.570 (*P* < 0.01), 0.950 (*P* < 0.01), 0.719 (*P* < 0.01) and 0.428 (*P* < 0.05), respectively, higher than for diseases of the circulatory system. Disease of the ear and mastoid process emerged as having a score lower than that of diseases of the circulatory system by 0.500 (*P* < 0.01).

**Table 4:** Results of linear regression analysis

Variables	Regression coefficient	Standard error	*t*	*P*	95%CI	
					Lower limit	Upper limit
Gender (control = female)						
Male	0.124*	0.065	-1.905	0.057	-0.004	0.252
Age group (control = youth)						
Middle age	0.128	0.086	-1.478	0.139	-0.042	0.297
Young elderly	0.572***	0.101	-5.657	< 0.001	0.373	0.770
Elderly	1.156***	0.138	-8.346	< 0.001	0.884	1.427
Long-lived elderly	3.138***	0.533	-5.893	< 0.001	2.094	4.182
Education level (control = elementary school and below)						
Junior high school	-0.038	0.081	-0.472	0.637	-0.196	0.120
Secondary or high school	0.115	0.129	-0.890	0.373	-0.139	0.369
College	-0.102	0.156	-0.655	0.512	-0.407	0.203
Bachelor’s degree and above	0.176	0.189	-0.928	0.353	-0.195	0.547
Marital status (control = never married)						
Married	0.047	0.151	-0.314	0.753	-0.248	0.343
Source of admission (control = non-emergency sources)						
Emergency sources	-0.103	0.134	-0.772	0.440	-0.366	0.159
Mode of admission (control = walk)						
Stretcher	2.252***	0.193	-11.689	< 0.001	1.874	2.630
Wheelchair	1.727***	0.145	-11.926	< 0.001	1.443	2.011
Purpose of visit (control = non-surgical)						
Surgical	0.294*	0.155	-1.9	0.057	-0.009	0.597
Occupation (control = farmer)						
Self-employed	-0.108	0.118	-0.914	0.361	-0.339	0.124
Retired (resigned) person	-0.305	0.192	-1.586	0.113	-0.682	0.072
Worker	-0.323**	0.162	-1.996	0.046	-0.640	-0.006
Other	-0.115	0.100	-1.150	0.250	-0.310	0.081
Medical payment method (control = new rural cooperative medical insurance)						
Basic medical insurance for urban residents	0.070	0.080	-0.873	0.383	-0.087	0.227
Basic medical insurance for urban workers	-0.012	0.125	-0.096	0.924	-0.257	0.233
Fully self-paid	-0.085	0.176	-0.484	0.628	-0.430	0.260
Other	0.231	0.340	-0.679	0.497	-0.436	0.898
Major diagnostic categories (control = diseases of the circulatory system)						
Certain infectious and parasitic diseases	-0.010	0.274	-0.036	0.971	-0.547	0.527
Neoplasms	0.752***	0.13	5.785	< 0.001	0.497	1.007
Diseases of the blood and blood-forming organs and certain disorders involving the immune mechanism	0.950***	0.357	2.663	0.008	0.250	1.649
Endocrine, nutritional and metabolic diseases	-0.117	0.096	-1.213	0.225	-0.305	0.072
Mental and behavioral disorders	0.207	0.315	0.657	0.511	-0.411	0.825
Diseases of the nervous system	-0.042	0.139	-0.303	0.762	-0.314	0.230
Diseases of the eye and adnexa	-0.259*	0.143	-1.813	0.070	-0.538	0.021
Diseases of the ear and mastoid process	-0.500***	0.114	-4.376	< 0.001	-0.725	-0.276
Diseases of the respiratory system	0.828***	0.209	3.955	< 0.001	0.417	1.238
Diseases of the digestive system	0.570***	0.111	5.119	< 0.001	0.352	0.788
Diseases of the skin and subcutaneous tissue	0.428**	0.214	1.997	0.046	0.008	0.849
Diseases of the musculoskeletal system and connective tissue	0.936***	0.187	4.995	< 0.001	0.569	1.304
Diseases of the genitourinary system	1.113***	0.164	6.801	< 0.001	0.792	1.434
Congenital malformations, deformations and chromosomal abnormalities	0.719***	0.274	2.625	0.009	0.182	1.256
Symptoms, signs and abnormal clinical and laboratory findings, not elsewhere classified	0.047	0.309	0.154	0.878	-0.558	0.653
Injury, poisoning and certain other consequences of external causes	0.615***	0.176	3.492	< 0.001	0.270	0.961
Factors influencing health status and contact with health services	0.837***	0.147	5.71	< 0.001	0.550	1.125
Intercepts	0.070	0.194	0.36	0.719	-0.310	-0.450

***P < 0.01, **P < 0.05, *P < 0.1

## Discussion

This study is the first on the needs relating to continuing care of rural patients in Mainland China. Based on the data available, it was found that the probability of needing a higher degree of continuing care after discharge was 3.25%. Patients were less prepared in terms of self-care/family’s caregiving ability and best prepared in terms of awareness and diet. Older patients with wheelchairs and stretchers had greater need for continuing care after discharge. In terms of major diagnostic categories, a greater need for continuing care following discharge emerged in the case of diseases of the genitourinary system, diseases of the musculoskeletal system and connective tissue, factors influencing health status and contact with health services, diseases of the respiratory system, neoplasms, injury, poisoning and certain other consequences of external causes, diseases of the digestive system, diseases of the blood and blood-forming organs and certain disorders involving the immune mechanism, congenital malformations, deformations and chromosomal abnormalities, and diseases of skin and subcutaneous tissue.

Readiness for hospital discharged was found to be related to patients’ age group, mode of admission, occupation, and major diagnostic categories. First, as patient’ age increases, readiness for discharge decreases, potentially as a result of the decline in the physiological functions of older patients. In this study, the older the age, the smaller the proportion of the age group. Paulina Hydzik et al. [26] concluded to a similar conclusion. Second, patients admitted in wheelchairs or on stretchers were less able to take care of themselves and experienced worse readiness for discharge than patients admitted on foot. In terms of occupation, workers had a slightly higher readiness for discharge than farmers, with no significant differences for other occupations. This may be explained in terms of the slightly greater levels of awareness of the importance of healthcare among workers. Providing support for this position, Zhang Xia et al. [27] conclude that degree of readiness for discharge is lower among patients engaged in fishery, forestry, agriculture and animal husbandry than among professional technical staff. Readiness for discharge can also be seen to be different for different diseases. Compared with the control group of diseases of the circulatory system, relatively poor levels of readiness for discharge are evident for patients with diseases of the genitourinary system, diseases of the musculoskeletal system and connective tissue, factors influencing health status and contact with health services, diseases of the respiratory system, neoplasms, injury, poisoning and certain other consequences of external causes, diseases of the digestive system, diseases of blood and hematopoietic organs and certain abnormalities involving immune functions, congenital malformations, deformations and chromosomal abnormalities, and diseases of skin and subcutaneous tissue. Patients with ear and mastoid disorders, on the other hand, appear to have experienced a higher level of readiness for discharge, most probably as a result of the characteristics and severity of the diseases such individuals suffer.

The result of the situation described above is that many needs for continuing care services remain unmet [28, 29], necessitating that more attention be paid to the needs of discharged patients in China and other LMICs. First, in the case of inpatients, caregivers can pay greater attention to older and/or stretcher- or wheelchair-admitted and farmer patients. It may also be beneficial to focus on patients with genitourinary disorders, diseases of the musculoskeletal system and connective tissue, factors affecting health status and excess to health services, diseases of the respiratory system, neoplasms, and other diseases with poor readiness for discharge, and to increase access to care services and educational guidance that would ideally be provided to patients during their stay in hospital. This would help to promote patient recovery, improve readiness for discharge, and reduce the need for continuing care services. Patients with scores greater than 8 are usually delirious or using tubes. On the basis of the above-mentioned relevant factors, if patients are also delirious or using the pipeline, then these patients will be the focus of attention.

Secondly, provide timely and appropriate continuing care after discharge. The perfect continuing nursing service can effectively alleviate the patient’s condition [30, 31]. According to the patients’ existing nursing problems, the corresponding nursing measures should be given to the patients after discharge. Universal services include systematic and comprehensive care assessment, as well as health education services. Targeted measures were provided, including replacement of gastric tubes and urinary catheters, control of infection, prevention of falls, frequency of patient care by patients’ families, regular blood pressure measurement, and timely back patting. China has established a long-term care insurance system, basically covering the above-mentioned content. However, there are differences in policy coverage among different regions due to the different speed of promotion. Therefore, we should further expand the scope of long-term care insurance pilot projects, optimize payment standards, and expand the coverage of the population. Improve pilot policies in a timely manner according to the results of pilot cities, and promote the implementation of long-term care insurance nationwide.

It is also suggested that there would be merit in strengthening publicity and education around continuing care services. Many patients continuing to have a low level of knowledge regarding continuing care services. Economic pressure can also inhibit the willingness of patients to obtain continuing care services, self-financed, retired patients with low demand for continuing care services [23].

There are some limitations. First, the data came from a local medical center. The medical service level of different regions in China is different. This conclusion should therefore be applied with caution. Second, there is no uniform continuous care needs assessment scale in China, this study uses RHDS approximate assessment. There is no assessment of the medical services received by patients after discharge, and the use effect of the RHDS after discharge needs to be further explored. Third, in some of the studies on discharge readiness, researchers used tools such as the activity of daily living to assess self-care ability, including information about potential care available to patients outside the hospital, such as the number of children. However, due to data limitations, this study does not contain such information.

## Conclusions

As with many LMICs, bridging the fragmentation of health services is a central task in the reform of China’s health care system, and the development of continuing care is certainly an important initiative. In China, some patients discharged from medical centers in rural areas experience a need for some form of continuing care. Establishing a supply system for continuing care service in rural areas as soon as possible and ensuring an appropriate point of interface with inpatient services may be an area of priority in terms of improving healthcare provision in rural China. This goal would also be furthered by the provision of long-term care insurance benefit packages that are appropriately complemented by the establishment of coverage for continuing care services in rural areas.

### Electronic supplementary material

Below is the link to the electronic supplementary material.

Supplementary Material 1

Supplementary Material 2

## Data Availability

The datasets generated and/or analyzed during the current study are not publicly available due to legal restrictions but are available from the corresponding author on reasonable request.

## Abbreviations

RHDS: Readiness for Hospital Discharge Scale
LMICs: Low- and middle-income countries
